# Periportal Paraganglioma: A Rare Cause of Obstructive Jaundice and Gastroesophageal Varices

**DOI:** 10.14309/crj.0000000000001742

**Published:** 2025-06-20

**Authors:** Irhoboudu D. Atogwe, Lefika Bathobakae, Fnu Deepali, Maria Lagarde Mussa, Olubunmi Shoyele

**Affiliations:** 1 Internal Medicine, Albert Einstein Medical Center, Philadelphia, PA; 2 Internal Medicine, St. Joseph's University Medical Center, Paterson, NJ; 3 Gastroenterology and Hepatology, Albert Einstein Medical Center, Philadelphia, PA; 4 Pathology and Lab Medicine, Einstein Healthcare Network, Philadelphia, PA

**Keywords:** paraganglioma, bile duct, periportal mass, obstructive jaundice, gastroesophageal varices, endoscopic ultrasound, portal vein stenting

## Abstract

Paragangliomas are rare neuroendocrine tumors arising from extra-adrenal chromaffin cells. Biliary tropism is exceptionally rare and can mimic primary biliary tract malignancies, thus presenting a diagnostic challenge. We describe a rare case of periportal paraganglioma presenting with obstructive jaundice and gastric and duodenal varices. Similar to other reported primary hepatic paragangliomas, this tumor was nonfunctioning, and there was no evidence of metastatic disease. The patient underwent portal vein stenting to relieve portal hypertension after failed laparoscopic resection because of high vascularity. He continues to follow-up with our clinic for serial blood tests and imaging and remains asymptomatic. This case highlights the importance of considering paraganglioma in the differential diagnosis of biliary tract masses.

## INTRODUCTION

Paragangliomas are rare neuroendocrine tumors that arise from extra-adrenal paraganglionic tissue.^1–3^ These tumors can be found in various locations throughout the body, most commonly in the head, neck, abdomen, urinary bladder, and para-aortic region.^4^ Biliary paragangliomas are very rare, and only a handful of cases have been reported to date.^5–8^We present an exceptionally rare case of periportal paraganglioma in a 29-year-old man with a history of ankylosing spondylitis. The patient presented with obstructive jaundice and was found to have a nonsecretory extrahepatic paraganglioma on imaging, which was confirmed by core needle biopsy. It is crucial to differentiate biliary tumors from extrahepatic nonfunctioning paragangliomas due to significant differences in their clinical management, prognosis, and genetic implications.

## CASE REPORT

A 29-year-old man with a history of ankylosing spondylitis treated with etanercept was referred to the gastroenterology clinic for elevated liver enzymes. The patient was started on etanercept therapy approximately 1 year before presentation and denied any side effects. During his visit, he denied headache, acute dysphagia, palpitations, diaphoresis, abdominal pain, pruritus, diarrhea, melena, recent weight loss, dark urine, or gross hematuria. The patient was a lifetime nonsmoker and denied the use of alcohol and illicit substances. Physical examination was notable for bilateral paraspinal tenderness, and vital signs were within the normal ranges. His most recent blood test results were significant for elevated levels of hepatic transaminases, alkaline phosphatase, and gamma glutamyl transferase (Table 1). Serological tests for ingestions, viral hepatitis, autoimmune disorders, and metabolic and genetic syndromes were unremarkable. Tumor markers and plasma metanephrine and normetanephrine levels were also normal.

**Table 1.** A summary table showing blood test results before and after endoscopic stenting

Laboratory parameter	On initial evaluation	After biliary duct stenting	Reference range
Alanine transferase	128	37	5–40 unit/L
Aspartate transferase	65	28	9–48 unit/L
Alkaline phosphatase	158	91	44–121 unit/L
Total bilirubin	1.1	0.9	0.0–0.3 mg/dL
Gamma glutamyl transferase	221	—	8–54 unit/L
Platelet count	137	—	150–400 K/uL
Total protein	7.2	—	6–8.5 mg/dL
International normalized ratio	1	—	0.851–1.14
Serum creatinine	1.01	—	0.5–1.5 mg/dL
Albumin	5.4	—	3.4–4.8 g/dL

**Figure 1.**
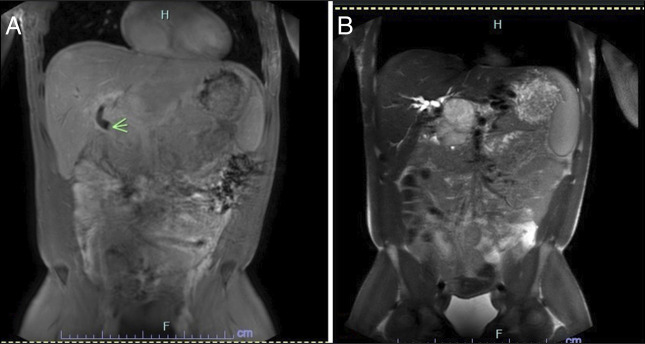
Magnetic resonance imaging of the abdomen images showing abrupt cutoff of the midcommon bile duct due to the periportal mass (green arrow).

An abdominal ultrasound (US) revealed several mass-like lesions throughout the right hepatic lobe, exerting a mass effect on the porta hepatis. A follow-up magnetic resonance imaging of the abdomen confirmed an external mass likely lymphoproliferative in origin, exerting pressure on the midcommon bile duct, with intrahepatic and extrahepatic biliary ductal dilatation (Figure 1). Compression of the hepatic vasculature led to the development of upper abdominal varices and collaterals in the porta hepatis. Notably, no morphological indications of cirrhosis or intraparenchymal liver lesions were observed.

**Figure 2.**
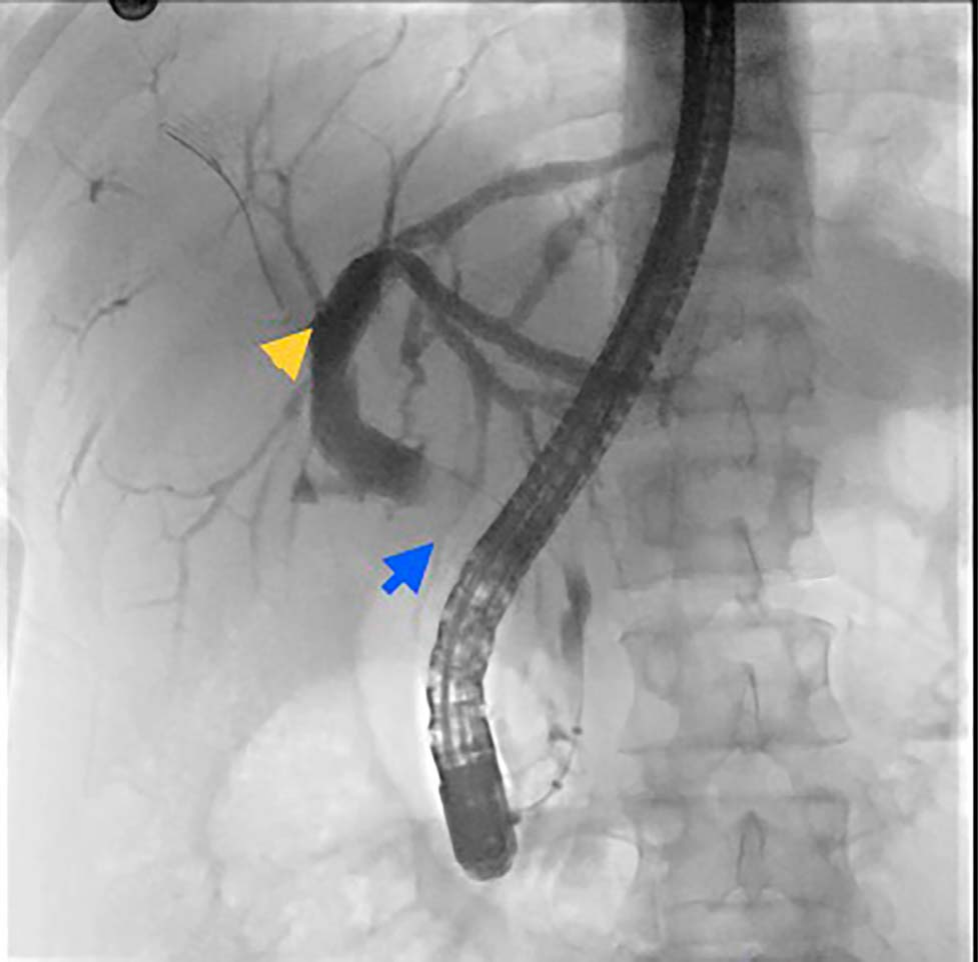
Endoscopic retrograde cholangiopancreatography image showing a filling defect (blue arrow) with common bile duct dilatation (yellow arrow).

An endoscopic US showed an 11 mm dilation of the common hepatic duct and a mass that was not amenable to biopsy due to increased vascularity. Subsequent endoscopic retrograde cholangiopancreatography demonstrated a 10 mm stenotic area in the main bile duct likely due to extrinsic compression by the porta hepatis mass (Figure 2). Brush cytology samples ruled out malignancy and a 10 Fr by 7 cm plastic biliary stent with a single external flap, and a single internal flap was inserted into the common bile duct to ensure patency. Liver enzyme levels normalized after stent placement. Esophagogastroduodenoscopy revealed small gastroesophageal and duodenal varices that did not require endoscopic intervention.

Given the complexity of the case and a high index of suspicion for a lymphoma, oncology service was consulted for comanagement. The diagnostic workup for lymphoma and other hematological disorders was normal. Scrotal US was negative for focal lesions, and staging computed tomography of the chest, abdomen, and pelvis was negative for metastatic disease. Although the positron emission tomography scan showed multiple areas of variable uptake, the radiologist concluded that they were artifacts given the recent use of contrast medium.

**Figure 3.**
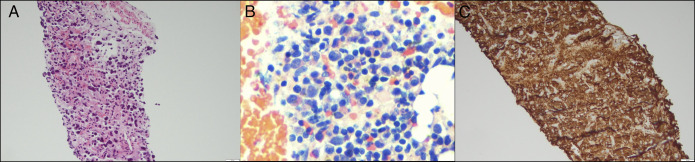
Histological and immunophenotypic profile of a periportal paraganglioma. Hematoxylin-eosin stains of the periportal mass (panel A, 20×). Note the epithelioid cells with eosinophilic cytoplasm, focal nuclear pleomorphisms, and nuclear hyperchromasia. Core needle biopsy reveals nests (zellballen pattern) of uniform round to oval cells with finely granular eosinophilic cytoplasm and centrally located nuclei with salt-and-pepper chromatin (panel B, 20×). The tumor cells are surrounded by delicate fibrovascular stroma. Immunohistochemical stains of the tumor cells are positive for chromogranin A (panel C, 20×) and synaptophysin, supporting neuroendocrine differentiation.

An interventional radiologist performed computed tomography-guided core biopsies of the periportal mass, and the histopathology was consistent with an extrahepatic paraganglioma (Figure 3). The neoplastic cells were strongly positive for synaptophysin, chromogranin, and GATA-3 (Figure 3). The cells were negative for CK7, TTF-1, CD45, CDX-2, CAM5.2, cytokeratin AEL/AE3, arginase, and CK20. S100 immunostaining was noncontributory, and the Ki-67 proliferative index was low. The patient was started on doxazosin and propranolol for catecholamine blockage and was referred to surgical oncology 2 weeks later for resection. Laparoscopic resection was aborted because of the high vascularity of the lesion, and portal vein stenting was pursued as a temporizing measure. Radiation therapy with cytoreductive resection was floated as an alternative treatment during the tumor board meeting. Although viable tumors located in other anatomic locations, there is a paucity of data relating to the safety and efficacy of external beam radiation therapy in extrahepatic paragangliomas. The patient continues to follow-up at our clinic with serial blood tests to monitor disease progression. He was also referred to the genetics clinic for counseling and possible testing. If the patient becomes symptomatic or the gastric and duodenal varices resolve, radiation therapy or a more radical resection may be considered.

## DISCUSSION

Paragangliomas can be sporadic or familial, with a significant proportion linked to genetic mutations, particularly in succinate dehydrogenase genes.^2,3,8^ Most of these tumors are diagnosed incidentally on imaging during investigations for unrelated complaints. However, about a third of the patients may present with signs of catecholamine excess, such as hypertension, palpitations, chest pain, excess sweating, myocardial infarction, anxiety, and headache.^3,4^ Obstructive jaundice is an extremely rare manifestation of paragangliomas and has only been observed in a few cases (Table 2).^5–8^ This unique presentation can pose a diagnostic challenge and predispose patients to significant perioperative morbidity and mortality.

**Table 2.** A summary table of published cases of primary hepatic paragangliomas

Author(s)	Year	Patient	Clinical presentation	Location	Treatment	Outcome
Caceres et al.^7^	2001	28/F	Abdominal pain	Common bile duct	Open cholecystectomy and Roux-en-Y hepaticojejunostomy	Cured with no evidence of tumor recurrence
Hitanant et al.^6^	1984	59/M	Obstructive jaundice	Common hepatic duct	Open surgical exploration	Cured with no evidence of tumor recurrence
Sarma et al.^5^	1980	37/M	Obstructive jaundice	Hepatic ducts	Exploratory laparotomy	Cured
Our case	2025	29/M	Obstructive jaundice	Porta hepatis	Biliary stenting, portal vein stenting after failed resection. Active observation	Currently asymptomatic

We describe an exceedingly rare case of extrahepatic paraganglioma presenting as obstructive jaundice and gastroesophageal varices. The patient first presented to our clinic with elevated liver enzymes and was found to have a periportal lesion with a mass effect on imaging. A biliary paraganglioma was confirmed on histopathology, but there was no evidence of metastatic disease or catecholamine excess. In symptomatic patients, the biochemical diagnosis of paragangliomas and pheochromocytomas begins with the detection of catecholamine metabolites (metanephrine and normetanephrine) in urine or serum plasma.^3,4^ Other markers include serum chromogranin A and methoxytyramine, which can be detected in metastatic disease.^3^ Although imaging plays a role in the diagnosis of paragangliomas, histopathology remains the gold standard for diagnosis.^3,4^

Surgical resection is the mainstay of treatment for localized and resectable tumors.^2,3^ The National Comprehensive Cancer Network (NCCN) guidelines recommend preoperative treatment with alpha-adrenergic blockage and fluid therapy to avoid hypertensive crises, malignant arrhythmias, and cardiovascular collapse during surgery.^2–4^ A laparoscopic approach is preferred when feasible to minimize perioperative morbidity. For metastatic or unresectable paragangliomas, other treatment options may include external radiation therapy with cytoreductive resection, immunotherapy, chemotherapy, and active observation.^2,9^

The management of biliary tract paragangliomas poses significant clinical challenges due to their rarity, vascularity, and proximity to critical hepatobiliary structures. In our case, the patient was started on medical therapy to prevent a catecholamine surge and subsequent hypertensive crisis. Endoscopic stenting was performed to relieve biliary obstruction, given the tumor's location encasing the common bile duct. After failed laparoscopic resection, the patient underwent endovascular vein stenting to treat the portal hypertension and varices. The patient is scheduled to follow-up with our service every 3 months for blood pressure monitoring and tumor marker checks as per the NCCN guidelines. For resectable tumors (paraganglioma/pheochromocytoma), the NCCN recommends follow-up within 12 weeks to 12 months of the surgery, then every 6 to 12 months for the first 3 years, and then annually for up to 10 years.^2^ After 10 years, follow-up and imaging are recommended when there is concern for cancer recurrence or metastases.

In summary, biliary paragangliomas are extremely rare neuroendocrine tumors that can present with obstructive jaundice. Although infrequent, these tumors may be considered in the differential diagnosis of biliary lesions as prompt recognition and intervention are crucial.

## DISCLOSURES

Author contributions: ID Atogwe conceptualized the idea of this case report. L. Bathobakae and F. Deepali assisted with literature search and drafting of the manuscript. ML Mussa and O. Shoyele edited, fact-checked, and proofread the final version of this case report. O. Shoyele prepared the pathology slides and interpretations. L. Bathobakae is the article guarantor.

Financial disclosure: None to report.

Previous presentation: This case was presented as an abstract at the American College of Gastroenterology (ACG) Annual Scientific Meeting, 2024 in Philadelphia, Pennsylvania. Atogwe, Irhoboudu D. MD,*; Mussa, Maria Lagarde MD, Barrett, Lisa DO, Echikunwoke, Blanche MBBS, Shoyele, Olubunmi MD. S2454 - A rare case of extrahepatic paraganglioma presenting with biliary obstruction in a 29-year-old man. *The American Journal of Gastroenterology* 119(10S): S1739, October 2024. DOI: 10.14309/01.ajg.0001039184.55876.80

Informed consent was obtained for this case report.

## ABBREVIATIONS:

US: ultrasound
NCCN: National Comprehensive Cancer Network
ERCP: endoscopic retrograde cholangiopancreatography
